# Supplementary figures and images for: ERK1/2-dependent TSPO overactivation associates with the loss of mitophagy and mitochondrial respiration in ALS
Source: Cell Death Dis. 2023 Feb 15;14(2):122. doi: 10.1038/s41419-023-05643-0 (PMC9931716; doi:10.1038/s41419-023-05643-0)

**Fig. 1A**

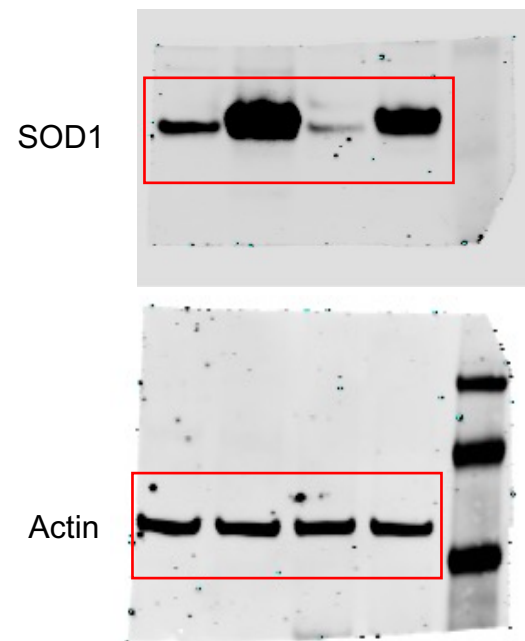

**Fig. 3B**

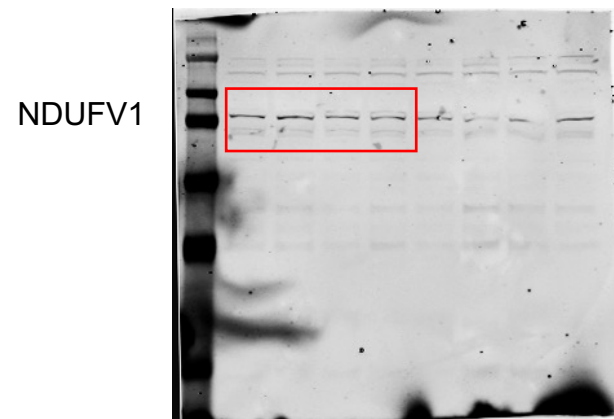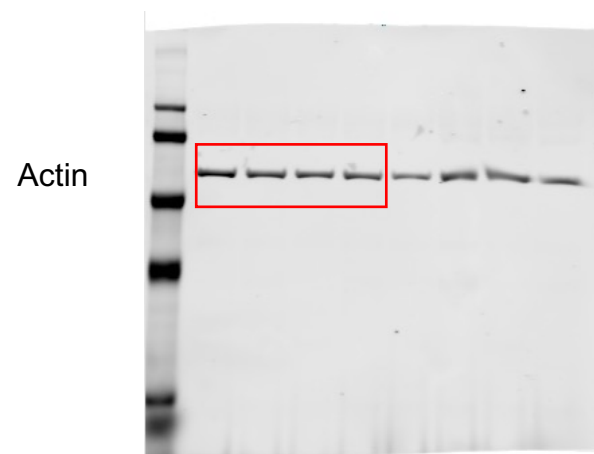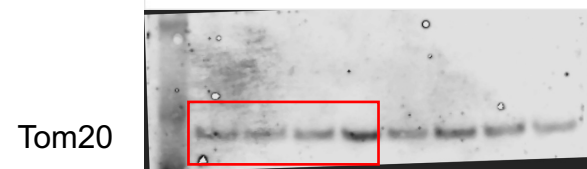

**Fig. 3D**

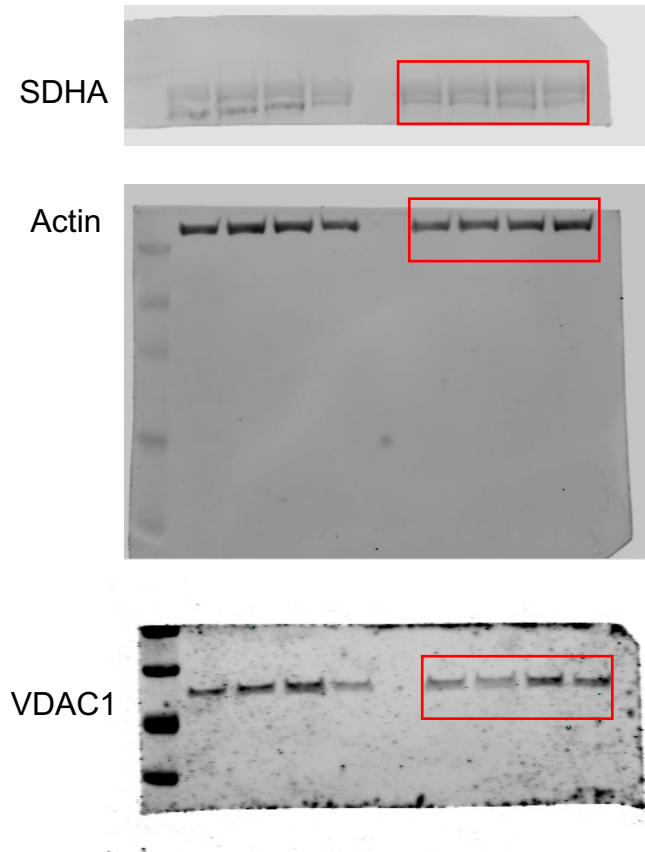

**Fig. 3E**

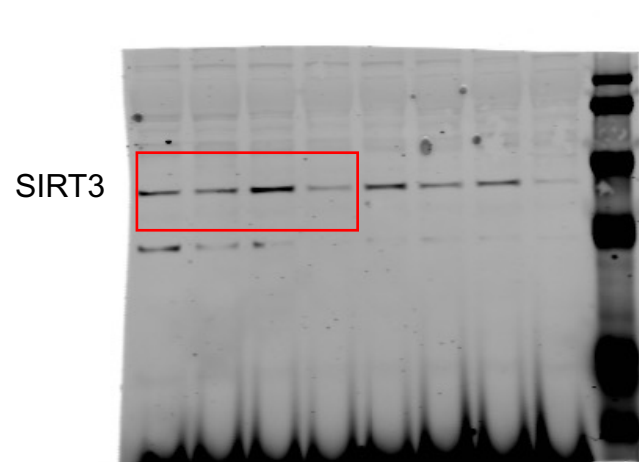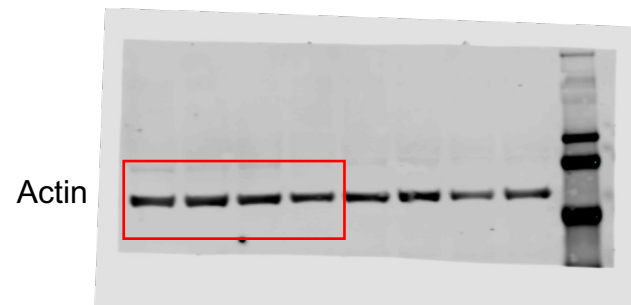

**Fig. 4A**

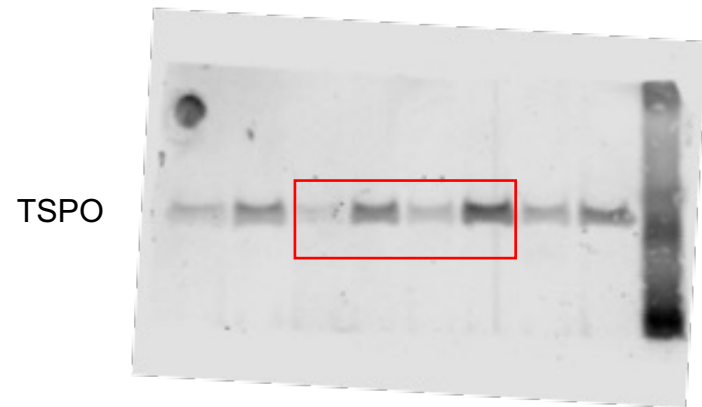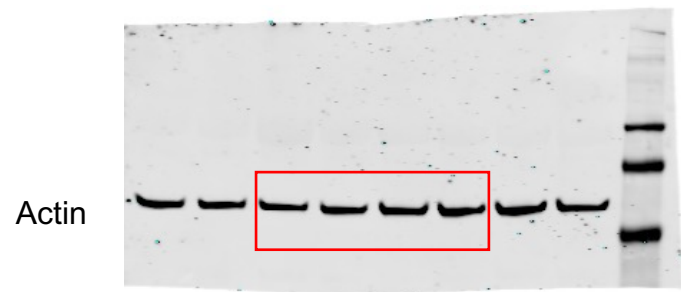

**Fig. 4B**

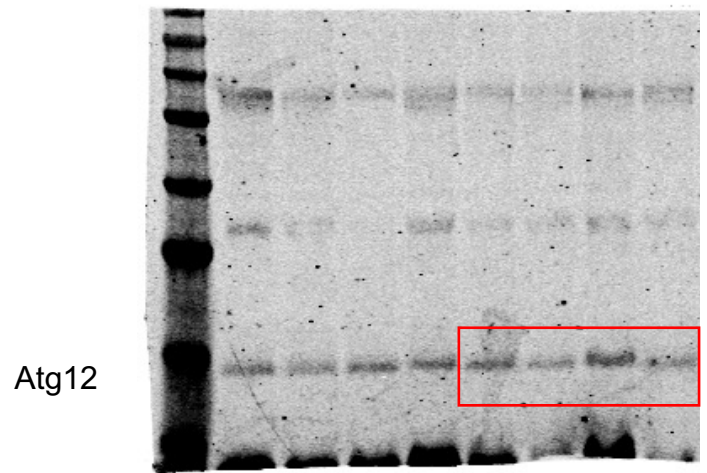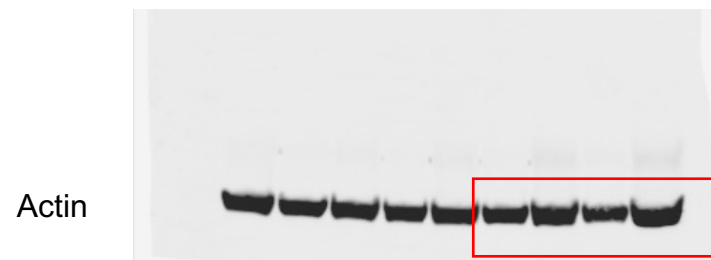

**Fig. 5A**

Total ERK1/2

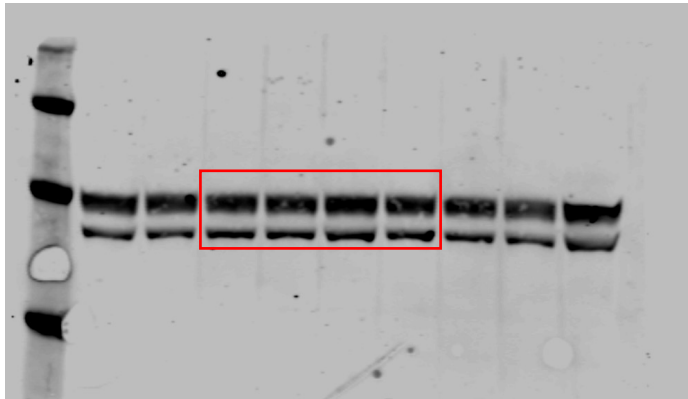

Phospho ERK1/2

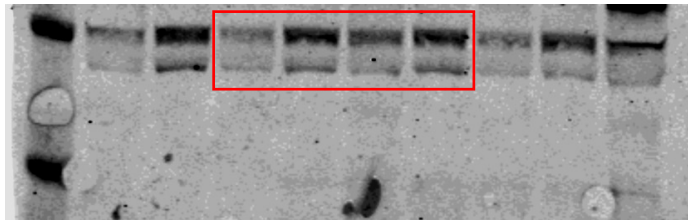

Total Stat3

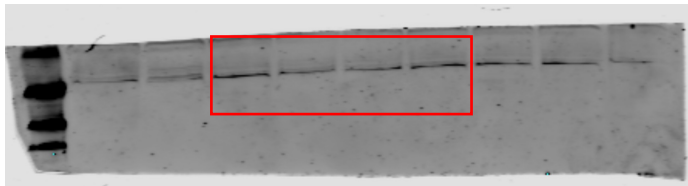

Phospho Stat3

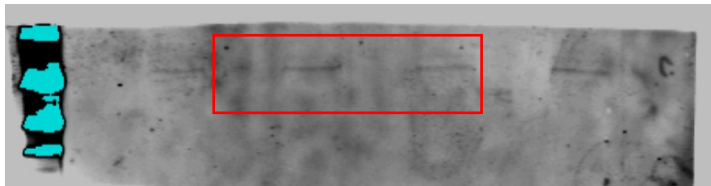

Actin

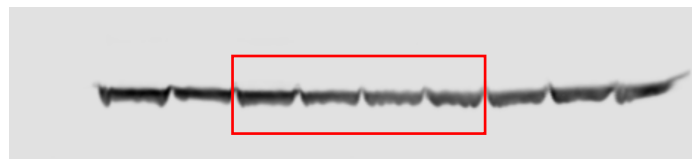

Supplement: Supplementary file 3 — Original Data File [file 41419_2023_5643_MOESM3_ESM.pdf]
